# Biocompatibility of hydrophilic silica-coated CdTe quantum dots and magnetic nanoparticles

**DOI:** 10.1186/1556-276X-6-299

**Published:** 2011-04-06

**Authors:** Jing Ruan, Kan Wang, Hua Song, Xin Xu, Jiajia Ji, Daxiang Cui

**Affiliations:** 1 National Key Laboratory of Nano/Micro Fabrication Technology, Key Laboratory for Thin Film and Microfabrication of Ministry of Education, Institute of Micro-Nano Science and Technology, Shanghai Jiao Tong University, 800 Dongchuan Road, Shanghai 200240, PR China

## Abstract

Fluorescent magnetic nanoparticles exhibit great application prospects in biomedical engineering. Herein, we reported the effects of hydrophilic silica-coated CdTe quantum dots and magnetic nanoparticles (FMNPs) on human embryonic kidney 293 (HEK293) cells and mice with the aim of investigating their biocompatibility. FMNPs with 150 nm in diameter were prepared, and characterized by high-resolution transmission electron microscopy and photoluminescence (PL) spectra and magnetometer. HEK293 cells were cultured with different doses of FMNPs (20, 50, and 100μ g/ml) for 1-4 days. Cell viability and adhesion ability were analyzed by CCK8 method and Western blotting. 30 mice were randomly divided into three groups, and were, respectively, injected via tail vein with 20, 60, and 100 μg FMNPs, and then were, respectively, raised for 1, 7, and 30 days, then their lifespan, important organs, and blood biochemical parameters were analyzed. Results show that the prepared water-soluble FMNPs had high fluorescent and magnetic properties, less than 50 μg/ml of FMNPs exhibited good biocompatibility to HEK293 cells, the cell viability, and adhesion ability were similar to the control HEK293 cells. FMNPs primarily accumulated in those organs such as lung, liver, and spleen. Lung exposed to FMNPs displayed a dose-dependent inflammatory response, blood biochemical parameters such as white blood cell count (WBC), alanine aminotransferase (ALT), and aspartate aminotransferase (AST), displayed significant increase when the FMNPs were injected into mice at dose of 100μg. In conclusion, FMNPs exhibit good biocompatibility to cells under the dose of less than 50 μg/ml, and to mice under the dose of less than 2mg/kg body weight. The FMNPs' biocompatibility must be considered when FMNPs are used for in vivo diagnosis and therapy.

## Introduction

Up to date, nanomaterials and nanotechnology have shown great potentials in disease diagnosis and therapy [1-7]. For example, a broad range of nanoscale inorganic particles including magnetic nanoparticles (MNPs) and quantum dots (QDs) have been systematically investigated for their unique physical, chemical properties, and their potential application in bio-detection, molecular imaging, and photothermal therapy of tumors [8-16]. Especially, MNPs have been used for magnetic resonance imaging (MRI), gene delivery, cell separation and cancer hyperthermia [17-19], and magnetic targeting, which provides an alternative method for targeted drug delivery systems to the desired location [20-22]. Up to date, QDs have been subjected to intensive investigations due to their unique properties and potential application prospect [23,24]. Several methods have been developed to synthesize water-soluble QDs for use in biological relevant studies [25,26]. For example, QDs have been used successfully in cellular imaging [27], immunoassays [28], DNA hybridization [29], optical barcoding [30], and drug carrier [31]. QDs provide a new functional platform for bioanalytical sciences and biomedical engineering.

In our previous study, we successfully prepared fluorescent MNPs composed of silica-coated CdTe QDs and magnetic nanoparticles (FMNPs), and used as-prepared FMNPs to label biomolecules for tumor imaging and hyperthermia therapy, and actively investigated their potential applications in bio-labeling, bio-separation, immunoassay, target imaging, and pathogenic detection [32-36]. Up to date, there were many reports which closely associated with preparation of fluorescent MNPs and their applications in biomedical engineering [37-47]. However, few report is closely associated with biocompatibility of fluorescent MNPs [48,49]. With the rapid development of nanotechnology, nanomaterials' biosafety has attracted more and more attention [50-56].

Herein, we investigated the effects of hydrophilic silica-coated CdTe QDs and magnetic nanoparticles (FMNPs) on human embryonic kidney 293 cells (HEK293) and mice with the aim of evaluating biocompatibility of prepared FMNPs. Our results showed that prepared FMNPs exhibited good biocompatibility under a special dose, and display great potential in applications such as in vivo molecular imaging, and hyperthermia therapy as well as in vitro pathogen separation and detection.

## Experimental

### Synthesis and characterization of FMNPs

FMNPs were prepared using improved method based on our previous reported method [40]. First, Fe_3_O_4_@Polystyrene (Fe_3_O_4_/PS) nanospheres were prepared according to Ref. [41], CdTe QDs were prepared according to our previous method [32], then CdTe QDs were modified to the surface of Fe_3_O_4_@Polystyrene (Fe_3_O_4_/PS) nanospheres, resultant CdTe QDs-covered MNPs were modified with silica shell using reverse microemulsion method, finally prepared FMNPs were washed with PBS (pH 7.4), isolated, and then saved for usage. Prepared FMNPs were characterized using scanning electron microscopy (SEM), high-resolution transmission electron microscopy (TEM, JEOL JEM2010, at 200 kV), the photoluminescence (PL) spectra (Perkin Elmer LS 55 spectrofluorimeter), and superconducting quantum interference device (SQUID) magnetometer (Quantum Design, PPMS-9T).

### Observation of cells incubated with FMNPs

HEK293 cells were seeded on cover slips for overnight, and were treated with medium with FMNPs (50 μg/ml) for 3 h. Then, the cells were rinsed with distilled water and stained with Prussian blue according to protocol. The HEK 293 cells were not treated with medium with FMNPs as the control. Then, the samples were attached to glass plates using mounting medium, and then were observed under an optical microscope (Olympus IX71).

### Fluorescent microscopy observation

HEK293 cells incubated with FMNPs (50 μg/ml) were collected, and fixed with 2.5% glutaraldehyde for 30 min, then, were incubated with 1 mM Hoechst 33258 in PBS (pH 7.4) for 5 min, and then were washed with PBS (pH 7.4) for three times, finally these cells were observed under fluorescence microscope (NIKON TS100-F).

### Flow cytometry analysis

HEK293 cells were treated without or with 20 μg/ml of FMNPs for 24 h, and were collected. After washing with PBS (pH 7.4), these cells were fixed with 70% ethanol/PBS for 30 min on ice. Approximately, 4 × 10^5 ^cells were centrifuged and re-suspended with PBS (pH 7.4), and then were analyzed by Calibur Flow Cytometers (BD Biosciences, Sunnydale, CA), the number of cells labeled with FMNPs were counted on.

### TEM observation of endocytosis course of FMNPs

HEK 293 cells were treated with 50 μg/ml FMNPs for 24 h. Then these cells were collected and fixed with 2.5% glutaraldehyde solution, and then embedded with epoxy resin, finally, these cells were made into the ultra-thin slices, and then observed with TEM.

### Effects of FMNPs on HEK 293 cells viability and proliferation assay

Effects of FMNPs on proliferation and viability of HEK293 cells were analyzed using Cell Counting Kit-8 (CCK8) assay. HEK293 cells were cultured in the 96-well microplate at the concentration of 5000 cells per well and incubated in a humidified 5% CO_2 _balanced air incubator at 37°C for 24 h. Except for control wells, the remaining wells were added into medium with FMNPs, final concentrations were, respectively, 20, 50, and 100 μg/ml, then those cells were continued to culture for 1-4 days, then, the ODs were measured using the Thermo multiskan MK3 ELISA plate reader according to the protocol of CCK8 assay kit, and calculated the survival rate of cells. The survival rate of cells can be calculated by the following equation:

### Detection of cell adhesion ability

Six-well plates were coated with fibrinogen (5 μg/ml) and vitronectin (1.5 μg/ml) in Dulbecco's Phosphate Buffered Saline (DPBS). HEK293 cells were harvested, washed for three times with serum-free minimal essential medium with Eargle's salt, and resuspended in attachment solution (calcium- and magnesium-free Hanks' balanced salt solution, 20 mM HEPES, 1 mg/ml heat-inactivated BSA, 1 mM CaCl_2 _and 1 mM MgCl_2_). Except for the control cells without treated with FMNPs, each well was added 1 × 10^4 ^HEK 293 cells and cultured for 1-4 days with FMNPs at different concentrations: 20, 50, and 100 μg/ml, unattached cells were washed with Hanks' balanced salt solution. The numbers of remaining attached cells after centrifugation were quantified spectrophotometrically at 405 nm for three times [55].

### Western blotting analysis of adhesive proteins

HEK293 cells were, respectively, incubated with FMNPs (20, 50, and 100 μg/ml) for 4 days, then they were collected and lysed in protein lysis buffer. Equal amounts of sample lysates were separated by sodium dodecylsulfate polyacrylamide gel electrophoresis (SDS-PAGE) and electrophoretically transferred onto polyvinylidene difluoride (PVDF) membranes (Millipore). The membrane was blocked with 0.1% BSA in Tris-Buffered Saline Tween-20 (TBST) buffer, and incubated overnight at 4°C with specific primary antibodies such as anti-fibronectin monoclonal antibody, anti-cyclin D3 antibody, anti-laminin antibody, anti-FAK antibody, and anti-β-actin antibody. Subsequently, the membrane was washed with TBST buffer and incubated with horseradish peroxidase-conjugated secondary antibodies. Enhanced chemiluminescence kits were used (Amersham, ECL kits) [55].

### Effects of FMNPs on mice

All animal experiments were performed in compliance with the local ethics committee. Kunming mice (female 28-30 g, 4-5 weeks old) were obtained from the Shanghai LAC Laboratory Animal Co. Ltd., Chinese Academy of Sciences (Shanghai, China) and housed in positive-pressure air-conditioned units (25°C, 50% relative humidity) on a 12:12-h light/dark cycle. The mice were allowed to acclimate at this facility for 1 week before being used in the experiment. All mice were divided into three test groups with FMNPs (20, 60, and 100 μg), and one control group (0 μg), and each mouse was injected with the suspension with FMNPs via tail vein. Those mice were, respectively, killed at No. 1-, 7- and 30-day post-injection, their organs such as heart, liver, spleen, stomach, kidneys, lungs, and brain were collected immediately. Then, those organs were fixed with 10% formaldehyde, embedded in paraffin, were cut into 20-μm section, stained with hematoxylin and eosin, and were observed by light microscopy. Three mice without FMNPs treatment were used as control.

After mice treated with different doses of FMNPs (20, 60, and 100 μg) for 1, 7, and 30 days, the blood were, respectively, collected in potassium EDTA collection tubes using a standard saphenous vein blood collection technique and were analyzed for standard hematology markers: red blood cell count (RBC), hemoglobin, hematocrit, mean corpuscular volume (MCV), mean corpuscular hemoglobin (MCH), mean corpuscular hemoglobin concentration (MCHC), platelet count, and white blood cell count (WBC). In order to separate serum, blood samples were centrifuged twice at 3000 rpm for 10 min. Liver function was evaluated with serum levels of total bilirubin levels (TBIL), alanine aminotransferase (ALT), aspartate aminotransferase (AST), and alkalinephosphatase (ALP). Nephrotoxicity was determined by blood urea nitrogen (BUN) and creatinine (Cr). The enzyme of lactate dehydrogenase (LDH) was assayed for evaluating cardiac damage. Albumin (ALB) was assayed as one parameter of damage of tissue or inflammation. These parameters were all assayed using a Hitachi 7600 Automatic Biochemical Autoanalyzer.

### Statistical analysis

Each experiment was repeated three times in duplicate. The results were presented as mean ± SD. Statistical differences were evaluated using the *t *test and considered significance at *P*< 0.05.

## Results and discussion

### Characterization of FMNPs

It is well known that MNPs can quench the fluorescent signal of QDs, which is because MNPs can absorb strongly the light emitted by QDs [42-44]. How to obtain FMNPs with high fluorescent signal and magnetic intensity is a great technical challenge. In order to prepare high performance of FMNPs, we improved the synthesis method based on our previous method [32]. We selected Fe_3_O_4_/PS nanospheres to replace Fe_3_O_4 _nanoparticles with the aim of reducing the QDs' fluorescence quenching caused by MNPs. The main procedure is as follows: first, fully mix Fe_3_O_4_/PS (100 nm average diameter) with CdTe QDs (3.5 nm average diameter) according to the ratio of 10:1, secondly, use ammonia-catalyzed hydrolysis method of tetraethyl orthosilicate (TEOS) to prepare silica-covered on the nano-composites composed of Fe_3_O_4_/PS MNPs and CdTe QDs. We optimized the FMNPs' reaction condition based on reverse micro-emulsion method through the Ternary phase diagram [48-50], finally obtained hydrophilic silica-coated CdTe QDs and MNPs with high fluorescent signal and magnetic intensity.

**Figure 1:**
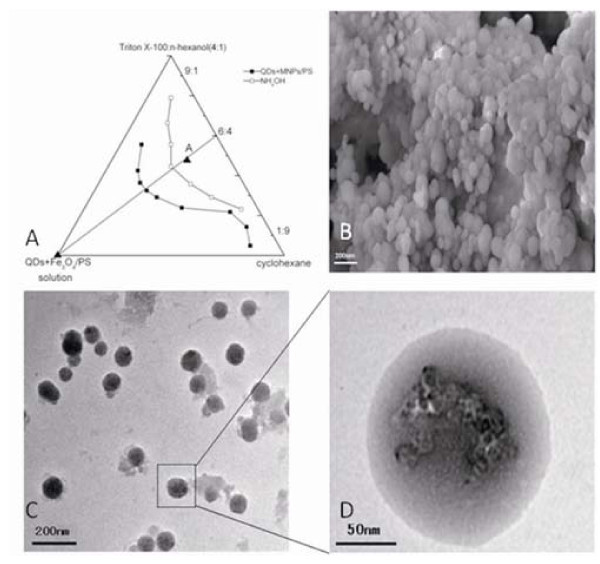
**Characterization of FMNPs**. **(a) **Ternary phase diagram of W/O reverse micro-emulsion reaction system; **(b) **SEM image of FMNPs, FMNPs were sphere with 150 nm in diameter; **(c) **TEM image exhibited that the FMNPs are mono-dispersed, silica shells covered Fe_3_O_4_/PS and QDs; and **(d) **the magnified image of the FMNPs presented a silica-shell thickness of 30 nm on average, and the mean total diameter was 150 nm.

As shown in Figure 1a, through measuring the conductivity and transparency of reverse micro-emulsion variations [48], we discovered that, under 6:4 of the surfactant (Triton X-100 and *N*-hexanol) and oil phase (cyclohexane), the micro-emulsion's maximal water solubilization had the wide range, thus, under above reaction condition, we can prepare the ideal FMNPs. Figure 1b showed the SEM images of FMNPs, prepared FMNPs were sphere with the average diameter of 150 nm. As shown in Figure 1c, d, FMNPs are mono-dispersed nano-composites composed of silica shells and Fe_3_O_4_/PS and QDs, the silica-shell thickness is almost 30 nm, the whole nanocomposites is almost 150 nm in diameter.

**Figure 2:**
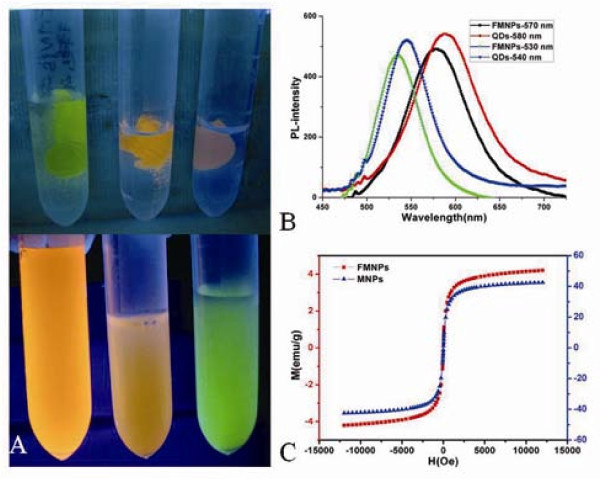
**The fluorescence intensity and saturation magnetization of the FMNPs**. **(a) **Images of FMNPs under UV irradiation with (top) and without (bottom) an external magnetic field; **(b) **PL spectra of different emission wavelength of CdTe QDs and FMNPs; **(c) **field-dependent magnetization curve of FMNPs at room temperature.

As shown in Figure 2a, FMNPs showed different colors under UV radiation, which is because we used different sizes of CdTe QDs in the course of preparing FMNPs. FMNPs were assembled and the solution became transparent under the external magnetic field (top), after removal of the external magnetic field, the aggregations were rapidly redispersed evenly (bottom). As shown in Figure 2b, the PL spectra of FMNPs showed that the emission peak of prepared FMNPs was symmetric with a 5 nm blue shift compared with pure QDs. As shown in Figure 2c, the magnetic hysteresis curve of prepared FMNPs clearly indicated that prepared FMNPs owned superparamagnetic property at room temperature with a saturation magnetization (*M*_s_) value of 4.0 emu g^-1^. This *M*_s _value was lower than the saturation magnetization of Fe_3_O_4_/PS (*M*_s _= 38 emu·g^-1^). The reduction of *M*_s _Value is attributed to the lower density of the magnetic component in prepared FMNPs.

### Localization of FMNPs inside cells

**Figure 3:**
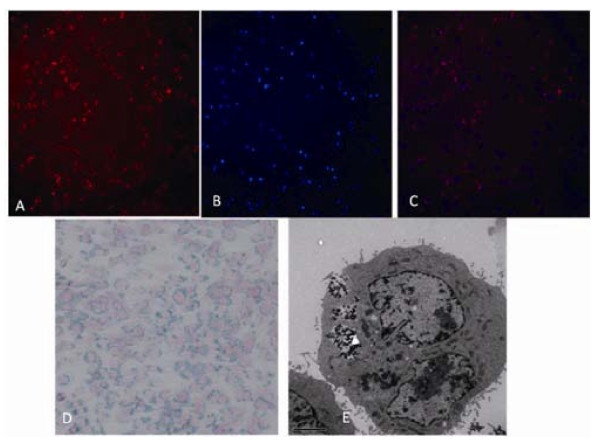
**Location of the FMNPs on HEK293 cell**: **(a) **the microscopy image of FMNPs presenting red fluorescence inside of the HEK293 cells; **(b) **the microscopy image of HEK293 cells nuclear counterstaining with Hoechst 33258; **(c) **image of combining the blue nuclear and red FMNPs; **(d) **the microscopy image of HEK293 cells' Prussian blue staining; and **(e) **TEM image of microstructure of HEK293 cell treated with 50 μg/ml FMNPs for 24 h.

As shown in Figure 3a, FMNPs were endocytosed by HEK293 cells, and located in the cytoplasm around nucleus, FMNPs exhibited red color (Figure 3a), and cell nucleuses exhibited blue color (Figure 3b), Figure 3c is the combined image of red and blue colors. The similar results were also confirmed by Prussian blue staining method (Figure 3d), and TEM imaging (Figure 3e). According to our observation, the HEK293 cells first formed pseudopodia, then wrapped FMNPs into cytoplasm, FMNPs mainly located inside lysosome and endosome [48,49]. Within cultured time, we did not observe that FMNPs were exited out of cells, FMNPs coexisted with cells well.

**Figure 4:**
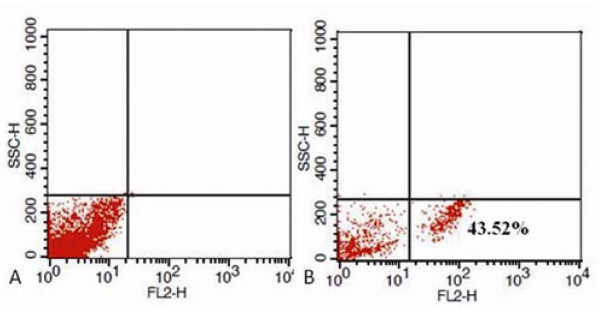
**Flow cytometry analysis the amount of HEK293 cells labeled with FMNPs**.

As shown in Figure 4, FCM analysis result showed that, HEK293 cells were treated with 20 μg/ml FMNPs for 24 h, the number of cells with FMNPs was 43.52% (right), the number of positive cells in control group is 0% (left).

### Effects of FMNPs on cell viability

**Figure 5:**
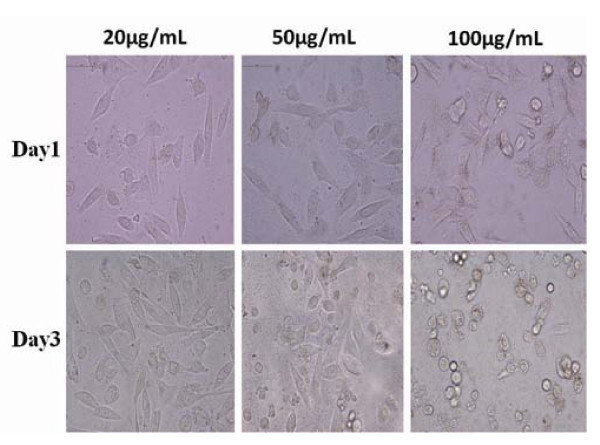
**Effect of FMNPs on HEK293 cells morphology (magnification = ×400), morphology of HEK293 cells cultured with different concentrations of FMNPs on different days, showing cells become round and floating with apoptotic characteristics**.

As shown in Figure 5, HEK293 cells were cultured with 50 μg/ml FMNPs for 72 h, HEK293 cells became round, and floated, apoptotic cells formed nodular structure encapsulated with FMNPs. When HEK293 cells were cultured with 100 μg/ml FMNPs for 72 h, these cells exhibited typical apoptosis features such as membrane vesicles, nucleus condensation, fragmentation, and apoptotic body formation. Within 50 μg/ml FMNPs in medium, HEK 293 cells grew well, FMNPs did not show toxic sign.

**Figure 6:**
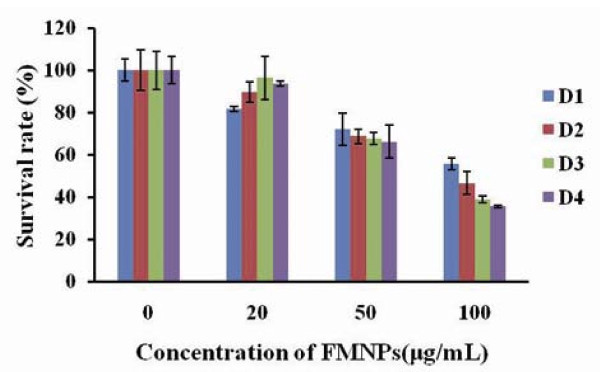
**Effect of FMNPs on survival rate of HEK293 cells, HEK293 cells viability measured by CCK8 assay and the percentage of cell viability was calculated as a ratio of OD of FMNPs-treated cells and control cells**.

Regarding the effects of FMNPs on HEK 293 cells, as shown in Figure 6, FMNPs affected the growth of HEK293 cells in time- and dose-dependent means, similar to our previous report [5,55]. FMNPs of 20 μg/ml in medium exhibited no toxicity to cells, the cell survival rate increased with the culture days increased. When the dose of FMNPs in medium reach or overrun 50 μg/ml, FMNPs exhibited low cytotoxicity to HEK 293 cells, the cell growth became slow. When the dose of FMNPs reach or overrun 100 μg/ml in medium, cell survival rate significantly decreased as the culture days increased. Thus, we consider that FMNPs exhibited good biocompatibility to HEK 293 cells within the dose of 50 μg/ml in medium.

### Effects of FMNPs on cell adhesion

**Figure 7:**
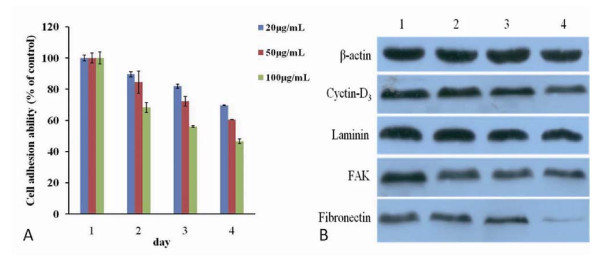
**Effects of FMNPs on cell adhesion ability and adhesive proteins**. **(a) **The adhesion ability of HEK293 cells treated with FMNPs. **(b) **Western blot analysis of the effect of FMNPs on the expressions of cyclin D3, laminin, FAK, fibronectin, and β-actin. Lanes 1, 2, 3, and 4 are the protein expressions at FMNPs concentrations of 0, 20, 50, and 100 μg/ml, respectively.

The adhesive ability of FMNPs-treated HEK293 cells were evaluated by centrifugation method. As shown in Figure 7a, the adhesion ability of HEK293 cells decreased gradually as the amount of FMNPs and culture days increased. As shown in Figure 7b, Western blotting analysis showed that the adhesive proteins such as laminin, fibronectin, FAK, and cell cycle protein cyclin D3 exhibited gradual down-regulation expression as the concentration of FMNPs increased. The β-actin protein expression as the control, kept unchanged in each case. These results showed that FMNPs can induce adhesion-associated proteins exhibit down-regulation expression, leading to thinning of cell basement membrane and decrease of the cell adhesion ability, finally resulting in cellular apoptosis or death.

### Effects of FMNPs on lifespan of mice

Regarding the effects of FMNPs on lifespan of mice, we used tail vein injection pathway to evaluate the in vivo toxicity of FMNPs. The mice were injected with 200 μl of FMNPs with the concentrations of 0 mg/ml (control group), 0.1 mg/ml (low dose group, LD), 0.3 mg/ml (medium dose group, MD), and 0.5 mg/ml (high dose group, HD). After cultured for 1 day, 1 week, and 1 month, the mice were killed by the method of cervical vertebra displace, and then used histopathology to evaluate inflammation degree of the mouse organs.

When the mice were injected with FMNPs of 20 and 60 μg, mice did not die, also did not show obvious clinical toxic signs, and then the body weight also increased accordingly. However, 3 of 9 mice injected with 100 μg FMNPs died (0 in the 1-day group, 2/3 in the 7-day group and 1/3 in the 30-day group). All mice deaths occurred in No. 1-30days after injection of the FMNPs. Before death, mice appeared lethargy, inactivity, and body-weight losses. In addition, the mice treated with 100 μg of FMNPs for 1day appeared weakness, and lost 10% of body weights within first week, these symptoms disappeared after 1week, mice could eat food normally, and their body-weight increased.

### Effects of FMNPs on important organs

**Figure 8:**
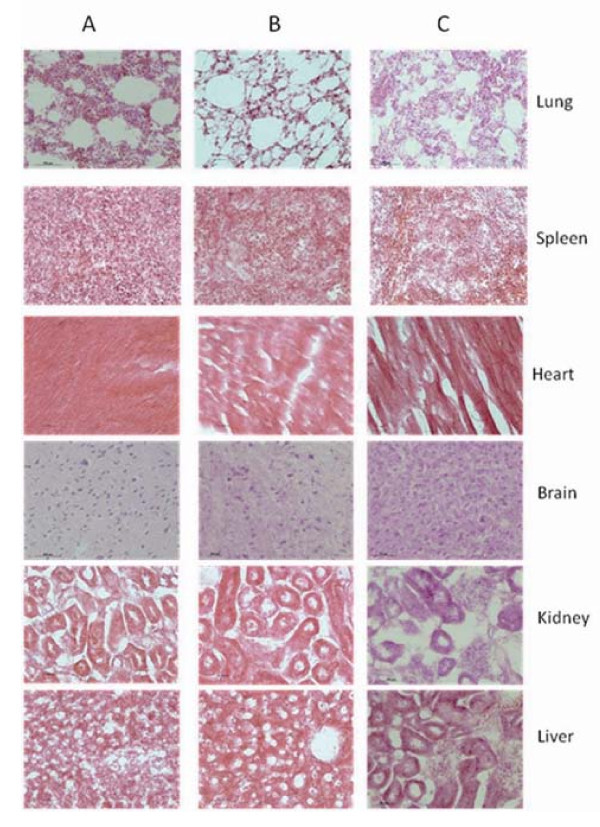
**The light micrograph of different organs about mice exposed to different dose FMNPs for 7 days**. **(a) **20 μg, **(b) **60 μg, and **(c) **100 μg (magnification=×400).

**Figure 9:**
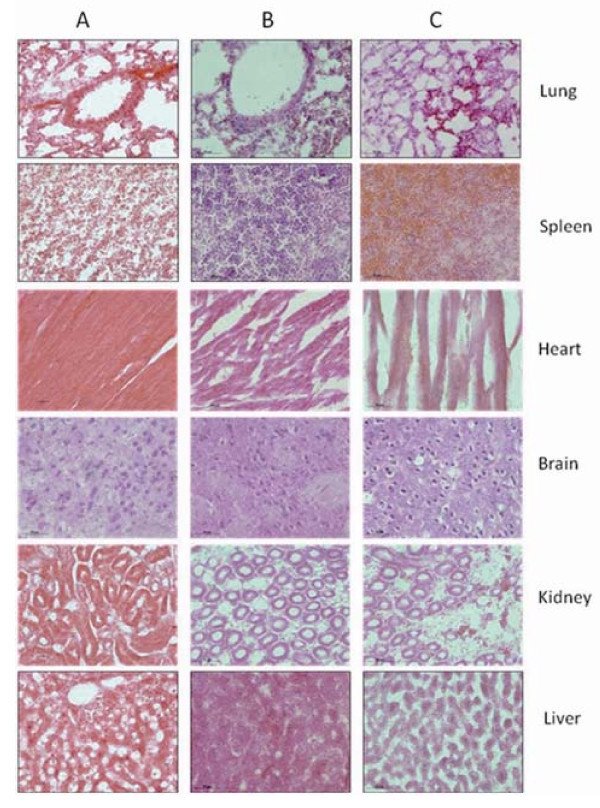
**The light micrograph of different organs from mice exposed to FMNPs of 60 μg at different exposure times**. **(a) **1day, **(b) **7days, and **(c) **30days (magnification=×400).

We also investigated the effects of FMNPs on important organs of mice. The pathological analysis showed that the FMNPs primarily accumulated in the lung, liver, and spleen, and pulmonary exposures to FMNPs exhibited a dose-dependent organ inflammatory response characterized by neutrophils and foamy alveolar macrophage accumulation. when the dose of injected FMNPs were lower than 60 μg, important organs did not exhibit obvious inflammatory response, conversely, when the dose of FMNPs overrun 60 μg, important organs appeared obvious morphological changes. Figures 8 and 9 showed that the morphological changes of different organs from mice exposed to different doses of FMNPs for 7 days, which clearly showed that some important organs such as liver, spleen, etc. appeared a dose-dependent series of granulomas. With the increase of dose of FMNPs, the toxicity reaction of mice became more and more severe. FMNPs induced mice to appear dose-dependent epithelioid granulomas and interstitial inflammation, large amount of inflammation cells infiltrated in lung alveolus interstitium, the alveolar septa became thicker and some lung alveoli were cracked.

**Figure 10:**
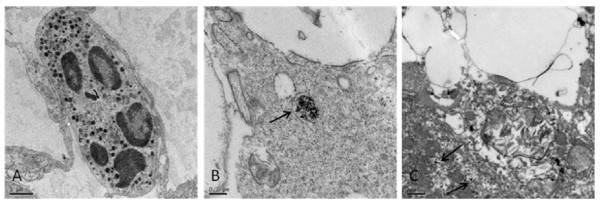
**TEM images of the ultra-structural features of the lung and spleen tissue exposed to FMNPs of 100 μg for 30 days**. **(a) **Lung tissue, **(b) **spleen tissue, and **(c) **liver tissue. FMNPs shown as arrow pointed.

In order to observe the high accumulation levels and to assess the biological effects of FMNPs in mice organs, ultrathin sections were prepared from the harvested mice lungs and liver for TEM imaging. Figure 10 showed the TEM images of the ultrastructural features of the lung (Figure 10a) and spleen (Figure 10b) tissues exposed to FMNPs. The FMNPs still remained in the lungs after 30 days, some in capillary vessel and some in cytoplasmic vacuoles of lung tissues. There were a lot of inflammation cell appeared in the wall of lung vacuole, such as multinuclear giant cells and acidophilic cells. The ultrastructural features of most cells appeared pathological changes. Thus, large dose of FMNPs can cause important organs of mice appeared obvious chronic toxic responses, low dose of FMNPs (less than 60 μg) almost did not cause obvious toxic signs.

Regarding the biodistribution of FMNPs in mice, as we observed, FMNPs mainly located in lung, liver, and spleen, no FMNPs was found in the brain tissues, which highly suggest that FMNPs cannot get through blood-brain barrier. Few FMNPs was observed in kidney of mice, which highly suggest that FMNPs is very difficult to be exited out by pathway of kidney, we speculate that FMNPs are mainly expelled out by liver secretion into bile tract system as shown in Figure 10c.

### Effects of FMNPs on blood biochemical parameters

**Figure 11:**
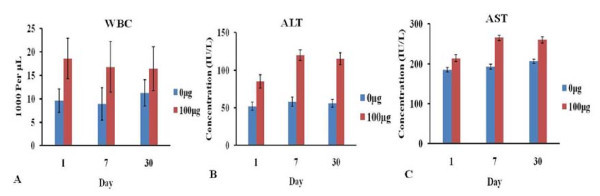
**Hematology and biochemical markers of mice after intravenous injection FMNPs**: **(a) **WBC, **(b) **ALT, and **(c) **AST.

We collected the blood samples post-injection on No. 1, 7 and 30 days and analyzed the blood markers. The hematological results indicated that measured factors were within normal ranges and there were not significantly different between groups with low and middle doses (20, 60 μg) and control (0 μg). Animals treated with higher dose (100 μg) showed significant increases in WBCs (*P*< 0.05) (Figure 11a), it illustrated that the higher dose (100 μg) of FMNPs could cause the inflammation reaction of mice.

Blood biochemical parameters that reflect the hepatic function and renal function were further investigated on No. 1, 7 and 30 days. Serum ALT or AST levels were increased with FMNPs' administration at 100 μg, while no increase was observed at 20 and 60 μg (Figure 11b, c). It illustrated that higher dose (100 μg, 2mg/kg body weight) of FMNPs influence the hepatic functions of mice. There were no significant changes for renal functions parameters such as urea nitrogen and others biochemical indexes after administration of FMNPs.

In this study, we also investigated the effects of as-prepared FMNPs on human fibroblast cells, gastric cancer MGC803 cells, and gastric mucous GES-1 cells, and obtained similar results, therefore, in this article, we did not display all data.

## Conclusion

FMNPs were successfully prepared by improved reverse micro-emulsion method. The prepared FMNPs possess strong fluorescent signal and high magnetization saturation intensity. FMNPs exhibited good biocompatibility to human normal cells with the concentration of 50 μg/ml FMNPs in cell medium, and to mice injected within the dose of 60 μg(2mg/kg. body weight) FMNPs. Conversely, when the dose of FMNPs reach or overrun 100 μg/ml in cell medium, or 60 μg to be injected into mice, cells and mice exhibited obvious toxic signs. Therefore, we strongly suggest that FMNPs can be safely used for cell labeling and in vivo tracking and imaging within 60 μg, and do not affect cell function and mice lifespan. Further work will focus on the surface modification of prepared FMNPs to enhance their biocompatibility. We believe that these prepared FMNPs have great potential in applications such as bio-labeling, bio-separation, immunoassay, in vivo targeting imaging and pathogenic detections.

## Abbreviations

ALT: alanine aminotransferase; ALB: albumin; ALP: alkalinephosphatase; AST: aspartate aminotransferase; BUN: blood urea nitrogen; Cr: creatinine; DPBS: Dulbecco's Phosphate Buffered Saline; Fe_3_O_4_/PS: Fe_3_O_4_@Polystyrene; HD: high dose; HEK293: human embryonic kidney 293; LDH: lactate dehydrogenase; LD: low dose; MNPs: magnetic nanoparticles; MRI: magnetic resonance imaging; MCH: mean corpuscular hemoglobin; MCHC: mean corpuscular hemoglobin concentration; MCV: mean corpuscular volume; PL: photoluminescence; PVDF: polyvinylidene difluoride; QDs: quantum dots; RBC: red blood cell count; SEM: scanning electron microscopy; SDS-PAGE: sodium dodecylsulfate polyacrylamide gel electrophoresis; SQUID: superconducting quantum interference device; TEOS: tetraethyl orthosilicate; TBIL: total bilirubin levels; TBST: Tris-Buffered Saline Tween-20; WBC: white blood cell count.

## Competing interests

In the past five years, all the authors haven't received any reimbursements, fees, funding, or salary from an organization that may in any way gain or lose financially from the publication of this manuscript, either now or in the future.

All the authors of this paper haven't hold any stocks or shares in any organizations that may in any way gain or lose financially from the publication of this manuscript.

All the authors of this paper haven't hold or applied any patents relating to the content of the manuscript, and all the authors haven't received reimbursements, fees, funding, or salary from any organizations that hold or have applied for patents relating to the content of the manuscript.

All the authors of this paper haven't any non-financial competing interests (political, personal, religious, ideological, academic, intellectual, commercial or any other) to declare in relation to this manuscript.

In conclusion, all the authors declare that no competing interests in this paper.

## Authors' contributions

JR and KW synthesized and characterized the materials, finished most of cellular and mice experiments, and drafted the manuscript. HS carried out partial bio-safety study, XX participated in the characterization of the materials and statistical analysis. JJ carried out the western blotting analysis. DC suggested the study, and participated in its design and directing to finish this study, and revising the manuscript. All authors of this paper have read and approved the final manuscript.
